# Effect of ultrasound combined with microbubbles therapy on tumor hypoxic microenvironment

**DOI:** 10.3389/fphar.2024.1502349

**Published:** 2025-01-13

**Authors:** Yuyi Feng, Danxia Qiu, Yangcheng He, Hai Jin, Liping Chen, Fen Xi, Zhiwen Hu, Yanlin Xie, Yucai Li, Minhua Lin, Pengxiao Sun, Yan He, Jianhua Liu

**Affiliations:** Department of Ultrasound, Guangzhou First People’s Hospital, South China University of Technology, Guangzhou, China

**Keywords:** tumor hypoxic microenvironment, ultrasound, microbubbles, hypoxia, oxygen partial pressure, contrast enhance ultrasound, blood perfusion

## Abstract

**Introduction:**

Tumor tissues exhibit significantly lower oxygen partial pressure compared to normal tissues, leading to hypoxia in the tumor microenvironment and result in resistance to tumor treatments. Strategies to mitigate hypoxia include enhancing blood perfusion and oxygen supply, for example,by decomposing hydrogen peroxide within the tumor. Improving hypoxia in the tumor microenvironment could potentially improve the efficacy of cancer treatments. Previous studies have demonstrated that ultrasound of appropriate intensity when combined with microbubbles, can improve tumor blood perfusion. However, its effects on tumor hypoxia remain unclear. This study aimed to assess the effects of low-frequency non-focused ultrasound combined with microbubbles at different intensities on tumor microenvironment hypoxia and to identify the optimal ultrasound parameters for alleviating tumor hypoxia.

**Method:**

Rabbits with VX2 tumors received ultrasound and microbubble treatments at different acoustic pressures and pulse repetition frequencies. The changes in tumor tissue blood perfusion before and after treatment were observed by contrast enhanced ultrasound (CEUS). The changes in tumor tissue hypoxia before and after treatment were observed by measuring oxygen partial pressure directly with in tumor tissue and immunohistochemical staining for hypoxia-inducible factor-1α (HIF-1α).

**Results:**

Results indicated that low frequency, non-focused ultrasound at 0.5 MPa/20 Hz and 0.5 MPa/40 Hz, when combined with microbubbles, could increase tumor tissue blood perfusion and improve the hypoxia in tumor tissues.

**Discussion:**

This study provides a new method for improving hypoxia in the tumor microenvironment (TME) which could potentially improve the cancer treatments resistance.

## 1 Introduction

The tumor microenvironment (TME) is a complex system that plays a crucial role in tumor development and significantly influences the effectiveness of tumor treatments (Wu and Dai, 2017). TME abnormalities include high interstitial fluid pressure, hypoxia, low pH, and other physicochemical characteristics (Spill et al., 2016). In most tumor regions, oxygen partial pressure falls below 5 mmHg, whereas normal tissues range between 30–60 mmHg (Jing et al., 2019; Brown and Wilson, 2004). Due to rapid cancer cell proliferation, nearly all solid tumors exhibit different degrees of hypoxia, leading to multiple effects such as chemotherapy and radiotherapy resistance, immune escape, reduced efficacy of photodynamic and sonodynamic therapies, and increased tumor invasiveness. Study indicates that tumor hypoxic microenvironment can be utilized for synergistic bioactivation of hypoxia-sensitive platesomes (Tao et al., 2022). Moreover, addressing hypoxia in the TME has become an essential focus in cancer therapy, as improving oxygenation within tumors could enhance treatment outcomes. Various methods are currently used to mitigate tumor hypoxia: these include directly increasing the oxygen supply via inhalation of 60% high-concentration oxygen (Hatfield et al., 2015), releasing oxygen from the oxygen-carrying microbubbles through ultrasound external irradiation (Ho et al., 2019), and increasing the tumor blood perfusion using mild hyperthermia or far-infrared photothermal therapy, both of which could enhance tumor blood perfusion and dilate tumor blood vessels (Chen et al., 2019; Jiang et al., 2019). Additionally, oxygen-generating nanomedicines have been developed to produce oxygen within tumors, alleviating hypoxia and enhancing subsequent therapeutic effects (Zhang et al., 2022; Liu et al., 2023). While each approach offers ways to improve the hypoxic tumor microenvironment, limitations exist. High-concentration oxygen therapy increases overall oxygen levels rather than specifically targeting hypoxic tumor regions. Similarly, mild hyperthermia applied through water bath heating increases systemic blood perfusion, limiting its specificity to tumor tissue. Although oxygen-carrying microbubbles can target tumors to release oxygen, the synthesis process is complex, and the amount of oxygen carried is relatively limited. Oxygen-generating nanoplatforms, while promising, face challenges in synthesis and limited penetration into tumor tissues. If ultrasound-stimulated microbubble cavitation (USMC) could improve tumor microenvironment hypoxia by increasing perfusion, it would provide a simpler, cost-effective means to enhance oxygen supply where it is most needed.

Research indicates that low-energy ultrasound combined with microbubbles can increase the permeability of tumor blood vessels, open the blood-tissue barrier, increase microcirculation and drug transport, and enhance therapeutic effects (He et al., 2024; Tang et al., 2023). The cavitation effect can improve vascular permeability and drug release while causing only mild vascular damage, which generally heals quickly (Chen et al., 2010; Lunt et al., 2008). Conversely, high-energy ultrasound can severely damage tumor blood vessels, significantly reducing or even blocking local blood flow (Xiao et al., 2020). Recent studies have shown that under low acoustic pressure (<1 MPa), ultrasound-stimulated microbubble cavitation (USMC) can enhance tumor blood perfusion, as seen in photothermal therapy, significantly transforming low-vessel tumors into relatively high-vessel tumors (Tang et al., 2023; Winslow et al., 2015). In contrast, high acoustic pressure (>2 MPa) can damage the tumor microvasculature, restrict tumor blood perfusion, and induce cell necrosis, potentially hindering drug delivery and worsening tumor hypoxia (Gao et al., 2016). The enhanced effect of USMC on tumor perfusion may present a novel approach to improving TME hypoxia and enhancing therapeutic outcomes.

Therefore, selecting optimal acoustic parameters for USMC to enhance tumor blood perfusion is thus likely to be a critical step in improving TME hypoxia. This research aimed to investigate the effects of different acoustic parameters of low-frequency, non-focused ultrasound combined with microbubbles on the hypoxic tumor microenvironment and to determine the most effective parameters for improving tumor hypoxia.

## 2 Materials and methods

### 2.1 Animals

Healthy female New Zealand white rabbits, each weighing approximately 2.5 kg, were obtained from the Guangdong Medical Laboratory Animals Center in Guangdong, China. Before tumor inoculation, all rabbits were acclimated for at least 7 days at a temperature of 24°C–26°C and a humidity of 45%–55%. This study was conducted in accordance with the principles of the Basel Declaration and Recommendations of Guide for the Care and Use of Laboratory Animals published by the United States National Institutes of Health (NIH publication no. 85–23, revised 1996). The experimental protocol received approval from the Laboratory Animal Ethics Committee of South China University of Technology, Guangdong (reference number 20210113).

### 2.2 Models

VX2 tumor tissue samples were sourced from the Cell Repository at Sun Yat-sen University in Guangzhou, China. Tumor tissue was cut into 1 mm^3^ pieces and placed in a culture dish with physiological saline solution. It was then injected subcutaneously into the superficial muscles of the left hind limb. The experiment commenced approximately 10 days after tumor implantation until the tumor reached a size of approximately 10 mm.

### 2.3 Experimental protocol

Tumor-bearing rabbits were randomly assigned to nine groups (n = 5 per group): blank control (BC), simple ultrasound (US), simple microbubble (MB), 0.3 MPa/20 Hz, 0.5 MPa/20 Hz, 1.0 MPa/20 Hz,0.3 MPa/40 Hz,0.5 MPa/40 Hz and 1.0 MPa/40 Hz groups.

The rabbits were anesthetized with 2% Pentobarbital Sodium, and a heating pad was used to maintain body temperature. In the USMC groups, rabbits received an intravenous injection of 0.1 mL/kg of microbubble contrast agent (perfluoro propane, 2 μm in size, Department of Ultrasound, Xinqiao Hospital Affiliated to Third Military Medical University, Chongqing, China) through the auricular vein, followed by an immediate 2 mL saline flush. Tumors were then treated with low-frequency, non-focused ultrasound (Shenzhen Well.D Medical Electronic, Shenzhen, China) at varying acoustic pressures (0.3, 0.5, or 1.0 MPa) for 6 min with a pulse repetition frequency of either 20 Hz or 40 Hz, duty cycle of 0.4% and pulse emission rest intervals were set at 9 s and 3 s. In the US group, rabbits received intravenous injections of sterile saline solution and were treated with 0.5 MPa/40 Hz ultrasound for 6 min. In the MB group, rabbits were injected with the same volume of diluted lipid microbubbles and exposed to sham ultrasound for 6 min. The BC group received an intravenous injection of sterile saline solution, followed by sham ultrasound exposure for 6 min.

#### 2.3.1 Contrast-enhanced ultrasound (CEUS)

Tumor tissues were examined using CEUS (GE LOGIQ E9, Probe: ML6-15) before and after treatment. The time-intensity curve (TIC) was analyzed using built-in software to assess changes in tumor tissue perfusion. Under ultrasound guidance, point-to-point TIC curve analysis was performed on the oxygen partial pressure measurement area to study the effects of different acoustic parameter combinations on tumor tissue blood perfusion. TIC curve analysis was performed on the CEUS curve of the pO_2_ measurement area and the quantitative parameters of blood perfusion, peak enhancement (PE, PE = Peak intensity - Base intensity), and area under the curve (AUC) in the same location. Treatment resumed only after verifying the complete clearance of microbubbles from circulation using contrast mode, ensuring no interference with experimental results. This procedure was repeated following treatment.

#### 2.3.2 Tumor oxygen partial pressure measurement

Before the CEUS examination, a needle marker from the tumor tissue oxygen partial pressure measurement instrument was placed at the tumor periphery. Tumor oxygen partial pressure was then measured using a tissue oxygen partial pressure measuring instrument (FS02; Shanghai Tower Tech. Ltd.) and an optic fiber oxygen sensor (PreSens, Germany). As the central tumor region typically exhibits poorer blood perfusion and more pronounced hypoxia than the periphery, making it a relatively severe area of tumor hypoxia. We analyzed whether USMC could improve TME hypoxia by directly measuring the pO_2_ of the central tumor area before and after treatment and based on the changes in tissue pO_2_ before and after treatment. The tissue pO_2_ sensor, connected to the data collection and analysis system, was calibrated following the manufacturer’s instructions. The needle was placed horizontally at the tumor’s height, and under two-dimensional ultrasound guidance, it was advanced to the center of the tumor, pushed forward by 2 mm as a whole. Then, retract the needle tube 2 mm to expose the tissue pO_2_ sensor inside the needle tip, to reduce tissue pressure on the sensor. After the measurement curve stabilized, the average pressure curve reading within 10 s was considered the pO_2_ value in that area.

**FIGURE 1:**
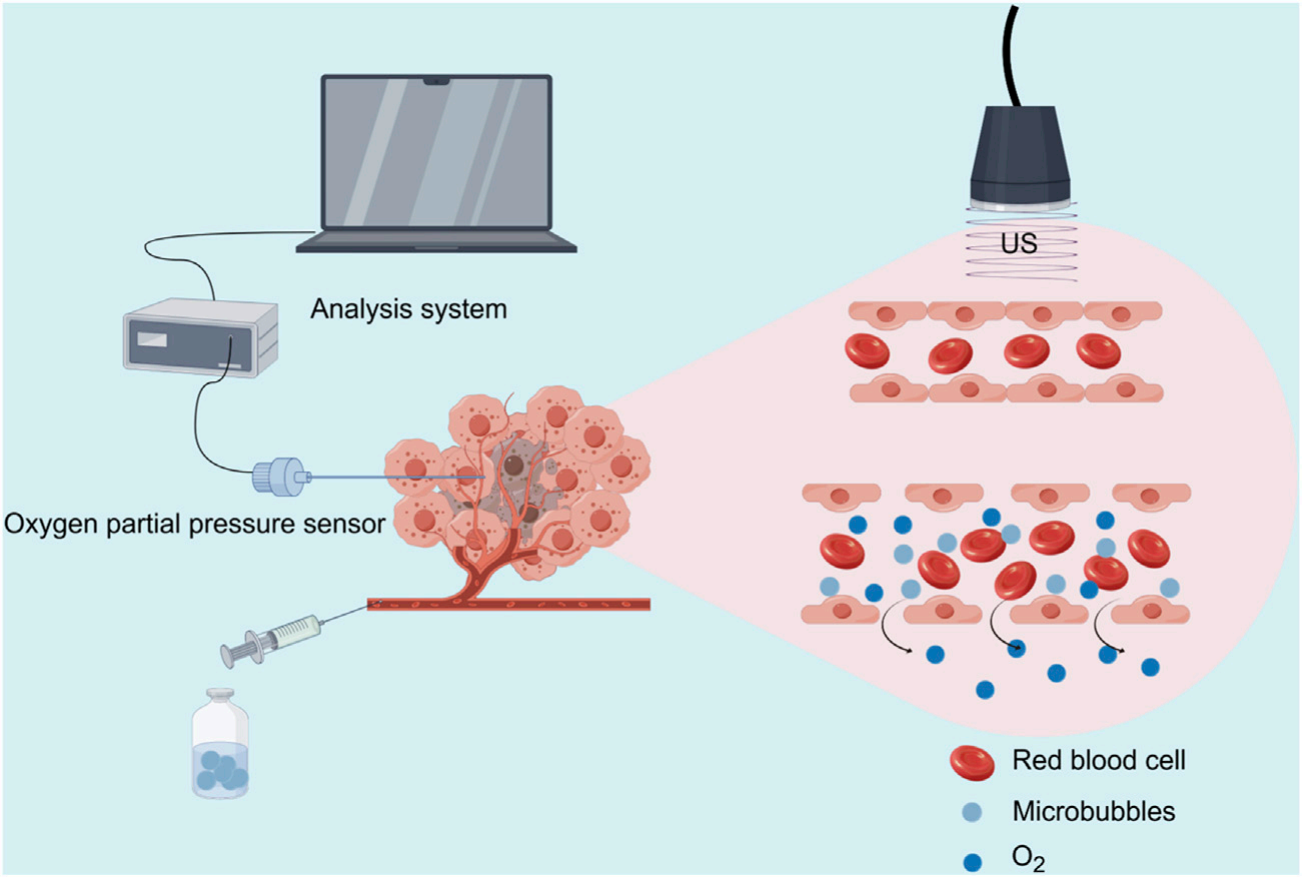
Schematic illustration of the USMC treatment and tumor oxygen partial pressure measurement (Source Figdraw).

During the treatment, the oxygen partial pressure sensor was retracted to the tip of the needle, and the entire device was maintained at the tumor periphery as a marker. Immediately after the treatment, tissue pO_2_ was re-measured in the same area under ultrasound guidance. Tumor tissue pO_2_ measurements before and after treatment were recorded using the Presens tissue pO_2_ experimental system. After CEUS examination and measurement of pO_2_ in the tumor tissues, each group was treated according to the corresponding conditions. After treatment, CEUS was performed, the partial oxygen pressure of the tumor was measured again, and the rabbits were sacrificed (Figure 1).

#### 2.3.3 HIF-1α immunohistochemical staining

To assess hypoxia changes in the TME post-USMC treatment, HIF-1α immunohistochemical staining was conducted. Rabbits were euthanized immediately after completing the measurement, and the tumor tissue was formalin-fixed, paraffin-embedded, sectioned, and blocked with 5% BSA for 30 min. Then, the slices were incubated with an anti-HIF-1α antibody (mouse monoclonal, 1:50, NOVUS 100–105) overnight at 4°C. After three PBS washes, the slices were incubated with Horseradish peroxidase-labeled anti-IgG antibody (1:500, Pinuofei) at 37°C for 1 h. The percentage of positive cells of immunohistochemistry staining slides in tumor tissues of each group was quantitatively analyzed by ImageJ. Five 40-fold magnifed felds of each group were randomly selected to calculate the percentage of positive cells, and the mean value was used as the quantified degree of tumor HIF-1α expression.

#### 2.3.4 H&E staining

Hematoxylin and eosin (H&E) staining was performed to evaluate any potential damage to the tumor tissue resulting from USMC treatment. After completing measurements, the rabbits were euthanized, and the tumor tissues were formalin-fixed, paraffin-embedded, sectioned, and stained with hematoxylin and eosin (H&E). Changes in tumor blood vessels and leakage of red blood cells were also observed. Pathological changes in the tumor tissue were examined under an optical microscope, and the corresponding images were captured.

### 2.4 Statistical analysis

Statistical analysis was conducted using GraphPad Prism 8.0 version, with measurement data presented as mean ± standard deviation. Sample groups were independent, with PE, AUC, and pO_2_ values considered as metric data following a normal distribution. The homogeneity of the PE, AUC, and pO_2_ values across the nine groups was tested using a fully randomized one-way ANOVA method. Paired sample t-tests were applied to compare PE, AUC, and pO_2_ values before and after treatment, with *p* < 0.05 indicating statistical significance. Before and after tumor treatment among different groups, Δ PE, Δ AUC, and Δ pO_2_ were used as measurement data, and a completely randomized one-way analysis of variance was used. The Pearson correlation coefficient was used for correlation analysis on Δ PE, Δ AUC and Δ pO_2_. The Bonferroni method was applied for multiple group comparisons, and an independent sample *t*-test was used for comparisons between two groups, with *p* < 0.05 denoting statistical significance.

## 3 Result

### 3.1 The changes in tumor microvascular perfusion before and after treatment through CEUS

#### 3.1.1 Visual evaluation

**FIGURE 2:**
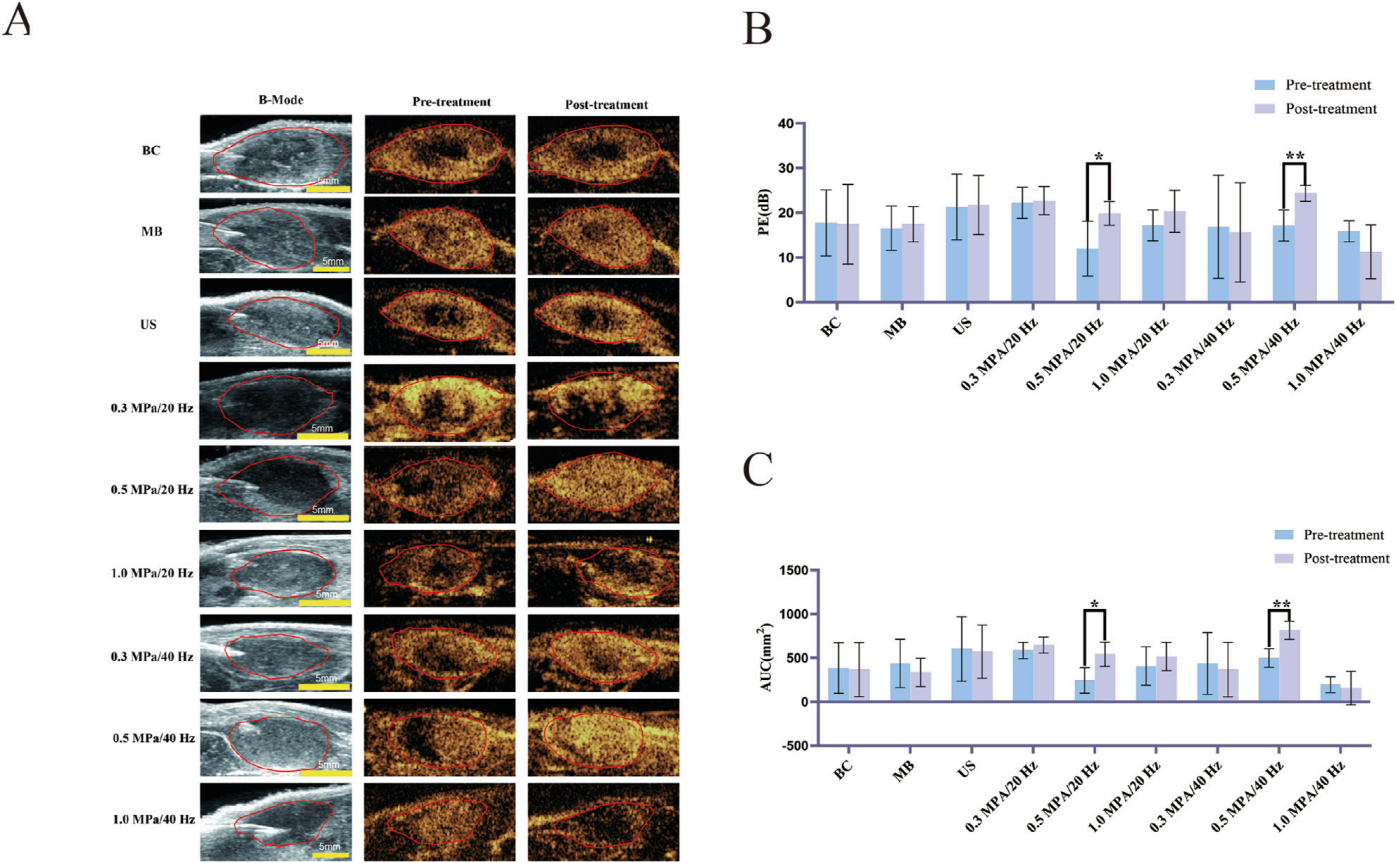
**(A)** Two-dimensional ultrasound images of tumors (B-Mode) and CEUS perfusion intensity before and after treatment in each group (n = 5). **(B)** The variation of Peak Enhancement (PE = Peak intensity - Base intensity) before and after treatment in each group (n = 5). **(C)** The variation of Area under the curve (AUC) before and after treatment in each group (n = 5). The error bar stands for standard deviation. Paired sample t-tests were applied to compare PE and AUC values before and after treatment (*p ˂ 0.05, **p ˂ 0.01).

Before treatment, CEUS imaging revealed no contrast agent filling or lower perfusion intensity in the tumor centers across each group than in the peripheral areas. Post-treatment, the 0.5 MPa/20 Hz and 0.5 MPa/40 Hz groups showed visibly increased perfusion intensity, with contrast agent filling defect areas. In contrast, the central filling defect areas in the 1.0 MPa/20 Hz and 1.0 MPa/40 Hz groups were enlarged, with no observed contrast agent entry. No significant changes were seen in the other groups (Figure 2A).

#### 3.1.2 CEUS quantitative analysis

Prior to treatment, there were no statistically significant differences (*p* > 0.05) in the Peak Enhancement (PE) or Area Under the Curve (AUC) values across tumor groups. Following treatment, PE values of the 0.5 MPa/20 Hz and 0.5 MPa/40 Hz groups increased significantly compared to baseline (*p* < 0.05). Although PE values in the 1.0 MPa/40 Hz group declined post-treatment, this change was not statistically significant. PE values in the other groups showed no significant changes (*p* > 0.05) (Figure 2B). AUC values in the 0.5 MPa/20 Hz and 0.5 MPa/40 Hz groups increased significantly after treatment (*p* < 0.05), while AUC values for other groups showed no significant changes (*p* > 0.05) (Figure 2C).

### 3.2 Tumor oxygen partial pressure

**FIGURE 3:**
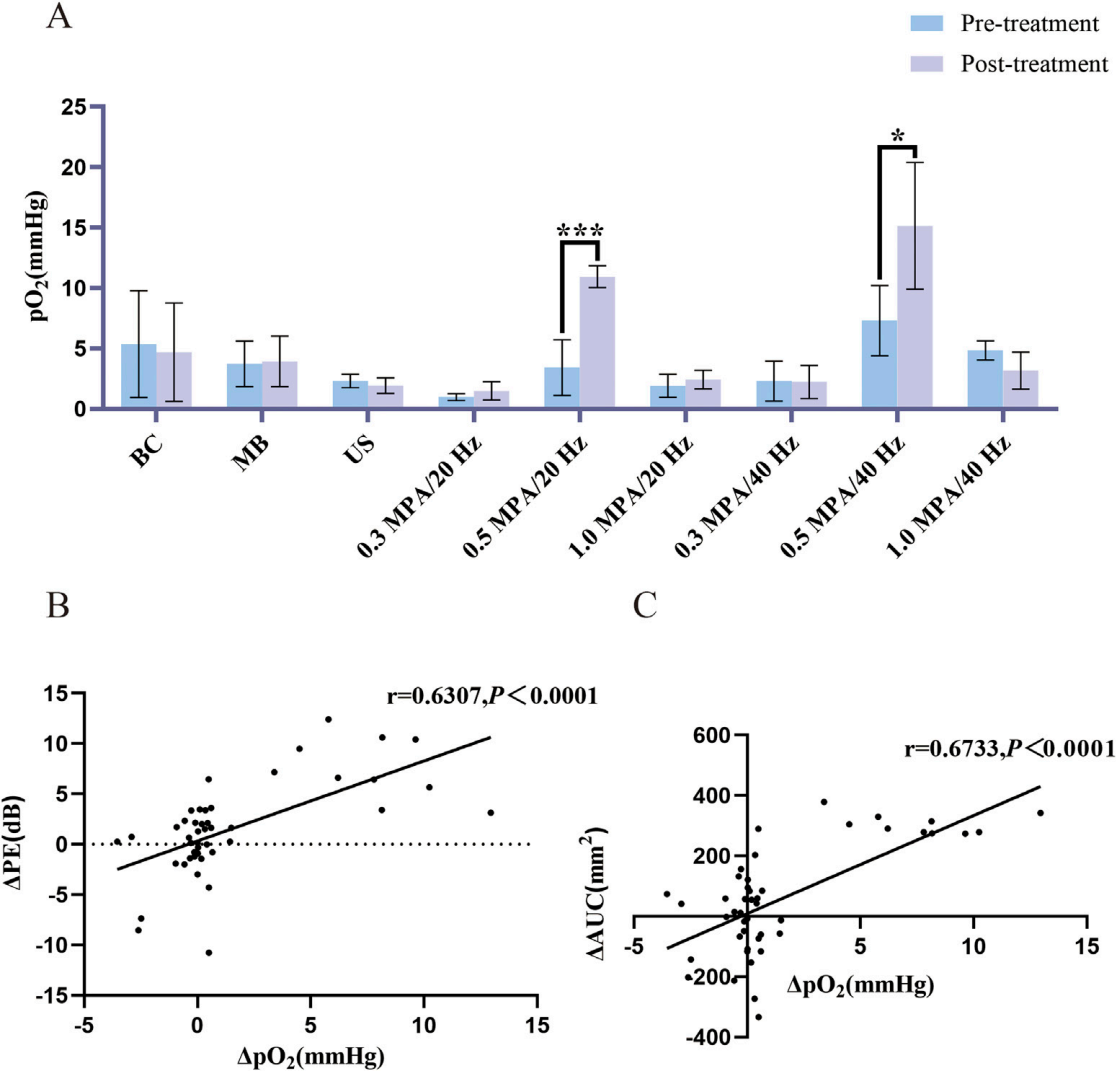
**(A)** Changes in pO_2_ before and after treatment (n = 5). Post-treatment, tissue pO_2_ levels in the 0.5 MPa/20 Hz and 0.5 MPa/40 Hz groups showed a statistically significant increase compared to pre-treatment levels. **(B)** Δ PE and Δ pO_2_ correlation analysis (n = 45). Δ PE and Δ pO_2_ were positively correlated. **(C)** Δ AUC and Δ pO_2_ correlation analysis (n = 45). Δ AUC and Δ pO_2_ were positively correlated. The error bar stands for standard deviation. Paired sample t-tests were applied to compare pO_2_ before and after treatment. The Pearson correlation coefficient was used for correlation analysis on Δ PE, Δ AUC and Δ pO_2_ (*p ˂ 0.05, ***p ˂ 0.001).

Before treatment, no statistically significant differences were observed (*p* > 0.05) in tissue pO_2_ levels among the groups. Post-treatment, however, tissue pO_2_ levels in the 0.5 MPa/20 Hz and 0.5 MPa/40 Hz groups showed a statistically significant increase compared to pre-treatment levels (*p* < 0.05). In contrast, there were no significant pO_2_ changes in the BC, US, and MB, 0.3 MPa/20 Hz, 1.0 MPa/20 Hz, 0.3 MPa/40 Hz, and 1.0 MPa/40 Hz groups before and after treatment (*p* > 0.05) (Figure 3A).

### 3.3 Immunohistochemistry (IHC)

**FIGURE 4:**
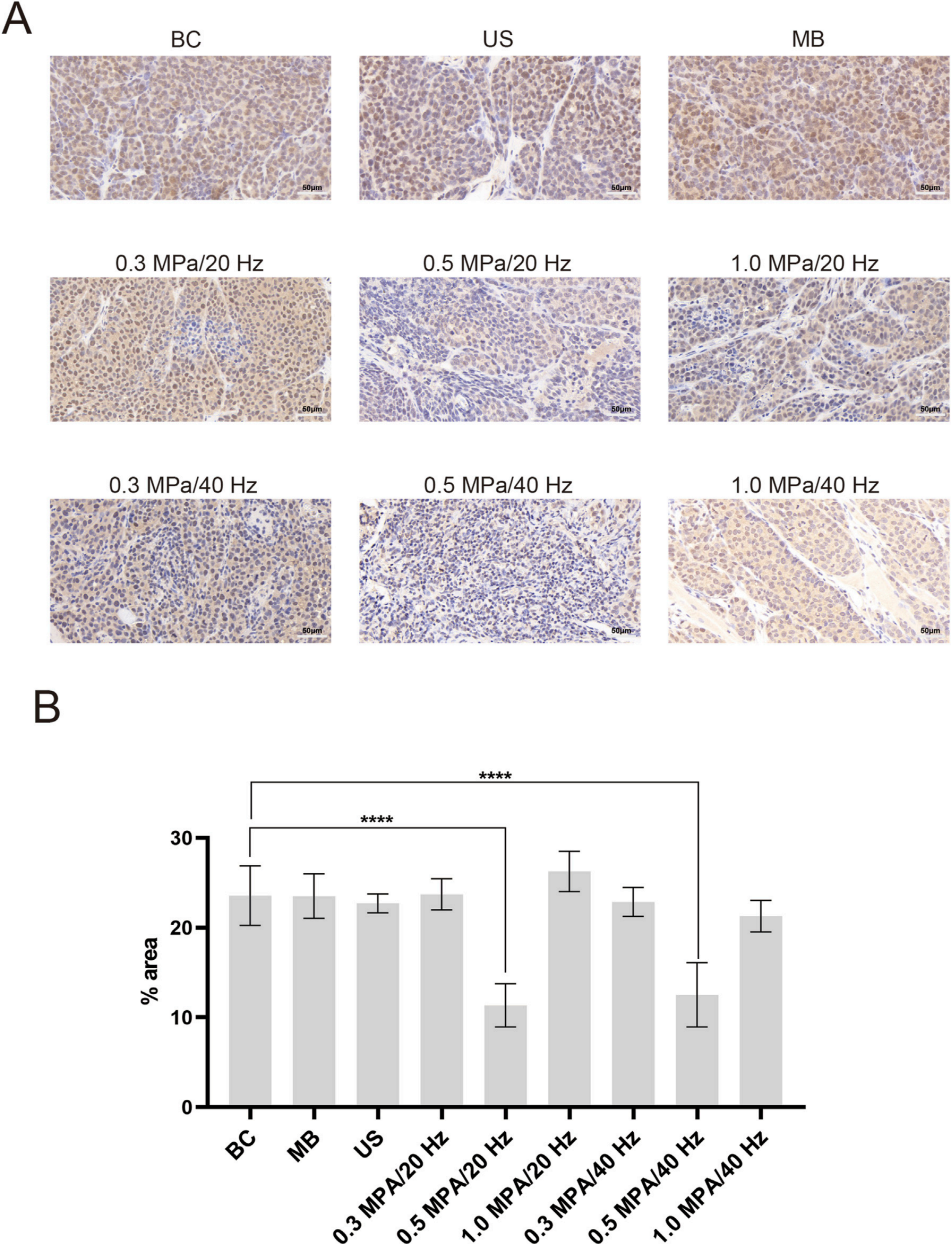
**(A)** HIF-1α immunohistochemical staining of tumor tissue of each group (n = 5). Compared with the other groups, the 0.5 MPa/20 Hz and 0.5 MPa/40 Hz groups showed a decrease in HIF-1α expression in tumor tissues (the brown substance in the nucleus and cytoplasm is HIF-1α positive expression). **(B)** Five 40-fold magnifed felds of each group were randomly selected to calculate the of the percentage of positive cells, and the mean value was used as the quantified degree of tumor HIF-1α expression. Repeat 3 times per group. The Bonferroni method was applied for multiple group comparisons. There was a statistically significant difference (*p* < 0.05) between the 0.5 MPa/20 Hz and 0.5 MPa/40 Hz groups and the BC group. The error bar stands for standard deviation (****p ˂ 0.0001).

Immunohistochemical staining indicated HIF-1α positive expression, identified by the presence of brown staining in the nucleus and cytoplasm of VX2 tumor cells. Results showed that all nine groups of tumor cells were positively expressed. Notably, the expression of HIF-1α in tumor tissue was decreased in the 0.5 MPa/20 Hz and 0.5 MPa/40 Hz groups compared to the other groups (Figure 4A). Quantitative analysis of the percentage of positive cells in each group using ImageJ software showed that there was a statistically significant difference (*p* < 0.05) between the 0.5 MPa/20 Hz and 0.5 MPa/40 Hz groups and the BC group (Figure 4B).

### 3.4 H&E staining

**FIGURE 5:**
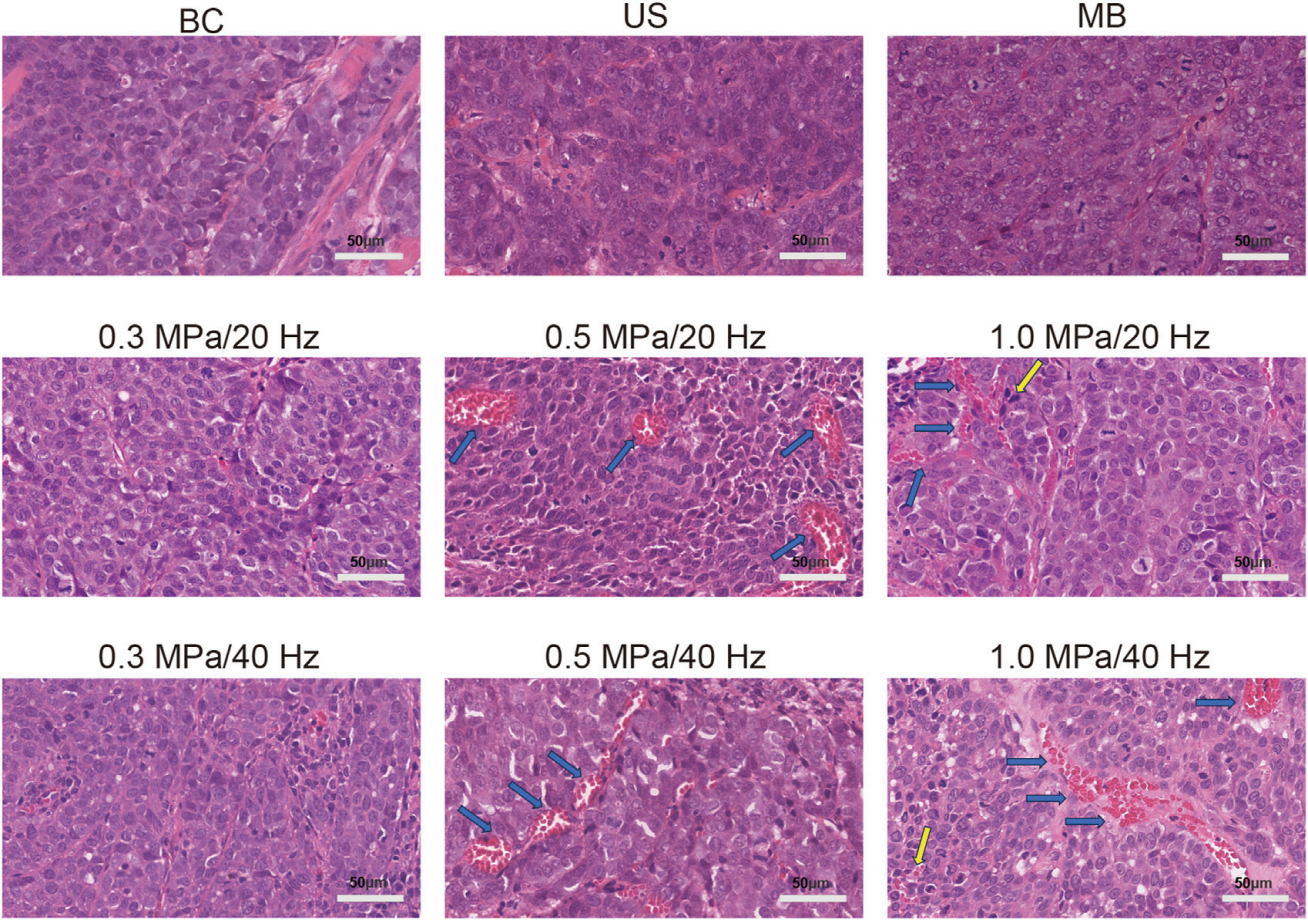
Vascular congestion (blue arrow) was observed in the 0.5 MPa/20 Hz and 0.5 MPa/40 Hz groups (n = 5), with continuous and intact vascular walls and red blood cells visible inside. Vascular congestion (blue arrow) was observed in the 1 MPa/20 Hz and 1 MPa/40 Hz groups, with most blood vessel walls intact and occasional scattered red blood cell leakage around the vessels (yellow arrow). The blood vessels in other groups showed no significant congestion, the vessel walls were intact, and no red blood cell leakage was observed.

Pathological sections of the tumor tissue were examined under an optical microscope, as shown in the figure. No significant tissue damage was observed in the blank control (BC), simple ultrasound (US), simple microbubble (MB), 0.3 MPa/20 Hz, 0.5 MPa/20 Hz, 1.0 MPa/20 Hz, 0.3 MPa/40 Hz, 0.5 MPa/40 Hz, or 1.0 MPa/40 Hz groups. Tumor cells were arranged in a cord-like pattern with large, deeply stained nuclei and prominent nuclear atypia. Vascular congestion was observed in the 0.5 MPa/20 Hz and 0.5 MPa/40 Hz groups, with continuous intact vascular walls and visible red blood cells. In the 1 MPa/20 Hz and 1 MPa/40 Hz groups, vascular congestion was also noted, with most of the blood vessel walls intact, though occasional red blood cell leakage was seen around the vessels. No significant congestion or leakage was observed in the remaining blood vessels across the other groups (Figure 5).

### 3.5 Analysis of quantitative changes in CEUS blood perfusion parameters (PE, AUC, and tissue PO_2_) before and after tumor tissue treatment

Compared to the control group, the Δ PE, Δ AUC, and Δ pO_2_ values in the 0.5 MPa/20 Hz and 0.5 MPa/40 Hz groups increased post-treatment, though no significant differences were observed in these two groups (Table 1). Δ PE and Δ pO_2_ were positively correlated (r = 0.6307, *p* < 0.0001) across all groups (Figure 3B). Similarly, Δ AUC and Δ pO_2_ also showed a positive correlation (r = 0.6733, *p* < 0.0001) (Figure 3C).

**TABLE 1:** The difference of the changes in PE, AUC, and pO_2_ between 0.5 MPa/20 Hz and 0.5 MPa/40 Hz groups (n = 5). ΔPE, ΔAUC and ΔpO_2_ presented as mean ± standard deviation.

Groups	0.5 MPa/20 Hz	0.5 MPa/40 Hz	F	*p*
ΔPE (dB)	7.862 ± 3.558	7.213 ± 2.993	0.329	0.763
ΔAUC (dB*s)	297.5 ± 23.96	315.8 ± 44.28	2.780	0.441
ΔpO_2_ (mmHg)	7.510 ± 1.553	7.848 ± 3.966	4.426	0.864

## 4 Discussion

Hypoxic regions within tumors are well-known contributors to resistance against chemotherapy, radiation therapy, and immunotherapy, as well as to tumor recurrence and metastasis (Sun and Lu, 2020; Grosser et al., 2019). Tumor blood perfusion plays a crucial role in the development of hypoxia within tumor tissue. Our previous study evaluated hypoxia in tumor tissues through blood perfusion. Given that contrast-enhanced ultrasound (CEUS) is a sensitive method for monitoring tumor blood perfusion, it can also be used to analyze tumor hypoxia. The effects of different acoustic parameters on tumor blood perfusion vary, with acoustic pressure being the most significant factor. Additionally, an increase in the pulse repetition frequency lowers the threshold for inertial cavitation (Lin et al., 2017). The appropriate intensity of USMC treatment has been proven to enhance tumor blood perfusion, but further research has not been conducted to determine whether the hypoxic conditions in the tumor tissue improve after increased perfusion. We hypothesized that USMC treatment could increase blood perfusion in hypoxic regions, potentially making them more responsive to treatment and improving patient prognosis. Therefore, we designed therapeutic ultrasound combinations with different acoustic pressures and pulse repetition frequencies. In this study, we assessed changes in tumor blood perfusion before and after treatment using CEUS quantitative parameters peak enhancement (PE) and area under the curve (AUC), which were derived from the time-intensity curve of CEUS. Additionally, tumor tissue pO_2_ levels were directly measured before and after treatment, and immunohistochemical staining was employed to observe HIF-1α in tumor tissue. Our aim was to explore whether changes in tumor blood perfusion induced by USMC alter the degree of hypoxia and identify the optimal acoustic parameter combinations for enhancing tumor blood perfusion and improving TME hypoxia.

We investigated the effects of low-frequency non-focused ultrasound combined with microbubbles at various acoustic pressures (0.3, 0.5, and 1.0 MPa) and pulse repetition frequencies (20 and 40 Hz) on tumor blood perfusion and hypoxia. We observed that USMC treatment at 0.5 MPa/20 Hz and 0.5 MPa/40 Hz increased tumor blood perfusion, as evidenced by an increase in the quantitative perfusion parameters, PE, and AUC, compared to pre-treatment values. Direct measurements of tumor tissue oxygen partial pressure and HIF-1α immunohistochemical staining revealed an increase in oxygen partial pressure and a reduction in tumor hypoxia following treatment at these parameters. Correlation analysis of tumor tissue oxygen partial pressure and the quantitative perfusion parameters showed a positive correlation between Δ PE and Δ AUC with Δ pO_2_. The pathological results for each group showed intact tumor blood vessels with no apparent defects in the vessel walls. After USMC treatment at the 0.5 MPa/20 Hz and 0.5 MPa/40 Hz groups, vascular congestion was observed, while in the 1 MPa group, a small amount of red blood cell leakage around the vessels was observed after treatment. Previous studies confirmed that USMC can lead to an increase in vasodilator substances, but the microvascular density of tumor tissue did not increase (Tang et al., 2023; Qiu et al., 2024). Therefore, it is inferred that the increased blood perfusion in tumor tissue is caused by USMC leading to tumor dilation. These findings suggest that USMC treatment improves tumor hypoxia by increasing tumor blood perfusion. Conversely, no significant changes in tumor perfusion were observed after USMC treatment at 0.3 MPa/40 Hz and 0.3 MPa/20 Hz groups, possibly due to insufficient cavitation effects caused by low intensity. At higher intensities (1.0 MPa/40 Hz and 1.0 MPa/20 Hz), CEUS imaging showed filling defects in the central region of the tumor, indicating a blockage in blood perfusion, likely caused by microvascular damage due to excessive intensity. After USMC treatment at 0.5 MPa/20 Hz and 0.5 MPa/40 Hz groups, there were no significant differences in Δ PE and Δ AUC between the two groups. Based on these findings, it can be reasonably concluded that the optimal acoustic parameters for enhancing tumor blood perfusion and improving the tumor hypoxic microenvironment were 0.5 MPa/40 Hz and 0.5 MPa/20 Hz.

Research has shown that after an appropriate intensity of USMC treatment, tumor blood perfusion increases for about 4 h (Tang et al., 2023). Therefore, the improvement of tumor hypoxia by USMC treatment only results in a transient increase in tumor tissue pO_2_, providing oxygen for subsequent treatment and enhancing the effectiveness of tumor treatment. Following USMC treatment, the gap between the tumor endothelial cells and blood perfusion increases, raising concerns about whether USMC treatment may promote tumor metastasis, which could be a significant safety issue (Snipstad et al., 2018; Gong et al., 2013). Zhang et al. treated rabbit VX2 tumors with two different USMC acoustic parameters combined with chemotherapy. The treatment group showed increased tumor perfusion compared to the control group, but there was no significant difference in metastasis to other organs (Zhang et al., 2021). The potential for increased tumor metastasis after USMC treatment may depend on the acoustic parameters used. Research has found that high acoustic pressure (5 MPa) increases the likelihood of melanoma metastasis compared to low acoustic pressure (2.1 MPa). However, this study removed the tumor 1 day after treatment and did not observe tumor metastasis over a more extended period (Douglas and Chunyan, 2006). Therefore, we reasonably inferred that whether tumor cells increase metastasis due to the biological effects of USMC may be related to the severity of cavitation effects. Low intensity USMC treatment primarily induces non-inertial cavitation in microbubbles, which does not damage the continuity of blood vessel walls. In contrast, high intensity USMC treatment can damage blood vessel walls, increasing the possibility of tumor cells metastasizing to other organs via the bloodstream.

The main limitation of this study is that it only demonstrated that USMC at an appropriate intensity can increase tumor blood perfusion and improve the tumor hypoxic microenvironment without assessing whether this prognosis of tumors would be improved due to the alleviation of tumor microenvironment hypoxia when combined with other treatments. The underlying mechanism by which USMC treatment regulates the hypoxic tumor microenvironment requires further investigation. Moreover, USMC is not an independent treatment but rather serves as a foundation for the immediate implementation of other therapies, such as chemotherapy and radiotherapy. Further research is needed to determine whether improving the hypoxic tumor microenvironment through USMC can enhance the therapeutic efficacy of antitumor drugs and other therapies targeting the hypoxic microenvironment.

## Data Availability

The original contributions presented in the study are included in the article/supplementary material, further inquiries can be directed to the corresponding authors.
